# Influence of Coagulation Factor VIII on Ischemic Stroke

**DOI:** 10.31083/RN44168

**Published:** 2026-01-26

**Authors:** María Del Mar Martínez-Salmerón, Laura Amaya-Pascasio, Patricia Martínez-Sánchez, Antonio Arjona-Padillo

**Affiliations:** ^1^Department of Neurology, Torrecárdenas University Hospital, 04009 Almería, Spain; ^2^Faculty of Health Science, Health Research Center (CEINSA), University of Almería, 04120 Almería, Spain

**Keywords:** biomarkers, blood coagulation, cryptogenic stroke, Factor VIII, ischemic stroke thrombophilia

## Abstract

**Background:**

There is considerable interest in the underlying mechanisms of cryptogenic stroke, with hypercoagulable states being widely studied. An elevated level of Factor VIII has been proposed as a potential prothrombotic marker associated with ischemic stroke. The aim of this study was to investigate the association between elevated Factor VIII levels and ischemic stroke and etiological subtype.

**Subjects and Methods:**

This retrospective observational study was conducted on subjects treated for ischemic stroke in the stroke unit of our institute between October 2018 and October 2023. Coagulative Factor VIII levels outside the acute phase (≥3 months) were measured, with elevated levels defined as >150%. Stroke etiologies (cryptogenic and non-cryptogenic: atherothrombotic, cardioembolic, lacunar, unusual, and coexistent causes), main cardiovascular risk factors, and prothrombotic biomarkers (protein C, protein S, antithrombin, anticardiolipin antibodies, anti-beta2-glycoprotein, lupus anticoagulant, and D-dimer) were recorded. Patients were categorized based on their level of coagulation Factor VIII (>150% vs. ≤150%). A comparative analysis was then conducted to assess differences associated with Factor VIII level.

**Results:**

A total of 68 patients were included, with a median age of 50.3 ± 12.2 years and a predominance of males (66.2%). The most frequent etiology was cryptogenic stroke (54.4%), followed by atherothrombotic (13.2%) and unusual causes (11.8%). Elevated Factor VIII levels were observed in 41.2% of patients. No significant associations were found between elevated Factor VIII and cryptogenic stroke (*p* = 0.27), stroke subtype (*p* = 0.38), comorbidities, or other thrombophilia biomarkers. However, a weak correlation was observed between elevated Factor VIII and antithrombin levels outside the normal range (*p* = 0.039), and a significant association was found between Factor VIII levels and prior atrial fibrillation (AF, *p* = 0.04).

**Conclusions:**

Although a high coagulation Factor VIII level was frequently observed in patients with ischemic stroke, this was not associated with cryptogenic stroke in the present cohort. Further studies with a larger sample size are warranted to clarify whether elevated Factor VIII is independently associated with ischemic stroke subtype, and whether elevated levels are a secondary finding related to inflammatory or systemic factors.

## 1. Introduction

Ischemic stroke is one of the main causes of disability and death worldwide. In recent years, its global incidence has increased due to the aging of the population and the increased prevalence of risk factors in certain populations [1]. Ischemic stroke is categorized into various subtypes according to its etiology, such as large-artery atherosclerosis, small-vessel occlusion, cardioembolic, or other known etiologies. If the cause is not identified after extensive investigation, it is referred to as cryptogenic ischemic stroke, or stroke of undetermined etiology. The latter is more common in young people [2] and includes the entity called ESUS (embolic stroke of unknown source) [3]. A large number of studies have been carried out on ESUS in recent years, mostly with negative results regarding the identification of its cardioembolic etiology and its treatment with anticoagulation. Cryptogenic ischemic stroke is a broader entity than ESUS, with potential causes involving stroke of cardiac, large- and small-vessel arterial origin, as well as hereditary or acquired hypercoagulability states. Coagulation Factor VIII has been investigated as a possible biomarker of hemostasis related to prothrombotic states.

Coagulation Factor VIII protein circulates in the body bound to von Willebrand factor and functions as an activator of Factor X in the coagulation cascade, resulting in the generation of fibrin thrombi. It is therefore a procoagulant cofactor and has previously been identified as a potential contributor to venous thromboembolism, myocardial infarction, and ischemic stroke [4]. However, its exact etiological role remains unknown, since it is also a biomarker of systemic inflammation [5] and hence its elevation may be partly in response to prior events. Moreover, other authors have observed persistent Factor VIII elevations lasting several years [6].

Elevated Factor VIII could be related to certain subtypes of stroke. Although the results are conflicting, it may be related to cardioembolic ischemic stroke [7], atherothrombotic stroke [8], and especially cryptogenic [9] strokes of unknown etiology. Finally, elevated Factor VIII has been associated with more severe presentations, greater neurological deterioration, worse functional outcomes, and higher recurrence rates [8].

Our initial hypothesis is that Factor VIII may be associated with ischemic stroke, particularly strokes of cryptogenic origin, and that its level is increased in this stroke subtype compared to others. The main objective of our study was therefore to evaluate the association between elevated Factor VIII, ischemic stroke, and the etiological subtypes in patients admitted to a stroke unit.

A better understanding of the relationship between elevated Factor VIII levels and ischemic stroke could have important implications for clinical practice. This may help to identify new causes of stroke, target secondary prevention more specifically, and provide more evidence of a possible etiological role for Factor VIII in ischemic stroke.

## 2. Materials and Methods

### 2.1 Study Design

This retrospective cross-sectional study was conducted at a University Hospital between October 2018 and October 2023. The hospital serves as a stroke referral center for a population of approximately 753,364 inhabitants. Data were collected from a prospectively maintained database of patients admitted to the stroke unit.

### 2.2 Patient Selection

Patients aged ≥18 years with a confirmed diagnosis of ischemic stroke and who underwent a complete etiological workup were included. Diagnostic evaluations followed standard institutional protocols, including medical history, physical examination, chest X-ray, ECG (electrocardiogram), neuroimaging (computed tomography [CT] and magnetic resonance imaging [MRI]), telemetry/Holter monitoring, transthoracic echocardiography, and neurosonological studies. When no definitive cause of stroke was identified with the standard cerebrovascular workup, an extended cardiological assessment was performed, including prolonged ECG monitoring and transesophageal echocardiography.

The diagnosis of ischemic stroke was confirmed by CT and/or MRI. Neurological evaluations were conducted by a neurologist from the stroke unit.

A laboratory test for potential underlying hypercoagulable states was requested via an arterial hypercoagulability screening panel. This was carried out according to the responsible physician’s criteria, based on the Spanish Society of Neurology guidelines [10]. Thrombophilia screening, including Factor VIII measurement, was not performed routinely, but only when clinically indicated. This was typically in younger patients (<50 years) and in patients without conventional vascular risk factors, with recurrent or multiple unexplained infarctions, a personal or family history of thrombosis, abnormal findings in routine laboratory tests, warfarin-induced skin necrosis (protein C or protein S deficiency), heparin resistance (antithrombin III deficiency), or clinical suspicion of antiphospholipid syndrome. Thrombophilia analysis included Factor VIII levels and was performed at least 3 months after the cerebrovascular event to avoid acute-stroke-phase alterations. Factor VIII levels were measured using a factor assay based on activated partial thromboplastin time (APTT). The functional capacity of this coagulation factor was evaluated by comparing the ability of standard and test plasma dilutions to correct the APTT time in Factor VIII-deficient plasma. The laboratory reference range was 50–150%.

The exclusion criteria included transient ischemic attacks (TIAs), hemorrhagic strokes, non-ischemic diagnoses, incomplete etiological studies, or thrombophilia workups performed during the acute phase (<3 months).

Between October 2018 and October 2023, a cross-search was conducted between the stroke unit registry—which included ischemic, hemorrhagic, transient ischemic strokes, and stroke mimics—and the hematology laboratory registry of patients who had undergone Factor VIII testing. Patients who met the inclusion criteria, appeared in both databases, and for whom Factor VIII measurements were made outside the acute phase were selected for this study. The selection process was limited to individuals for whom Factor VIII testing had been ordered. This may have introduced selection bias, as the decision to test was based on physician discretion.

### 2.3 Variables

Patients were categorized according to their coagulation Factor VIII level as either normal (50–150%) or elevated (>150%). Stroke etiology was classified using the Trial of Org 10172 in Acute Stroke Treatment (TOAST) criteria: (1) Large-artery atherosclerosis: significant (>50%) stenosis or occlusion of a major brain artery or branch cortical artery; (2) Cardioembolism: at least one known high- or medium-risk factor for cardioembolism; (3) Small-artery occlusion (lacunar): normal CT/MRI examination or a relevant brain stem or subcortical hemispheric lesion with a diameter <1.5 cm; (4) Stroke of other determined etiology (unusual cause): rare causes of stroke (stroke secondary to systemic disorders, such as connective tissue disease, infection, metabolic or coagulation disorders, or other vascular diseases such as arterial dissection, saccular aneurysm, arteriovenous malformation, etc., or migraine-related infarction; and (5) Stroke of undetermined etiology: cryptogenic (the cause of stroke cannot be determined with any degree of confidence), or undetermined by coexistent causes. Cryptogenic stroke was defined as a stroke of undetermined cause, including both ESUS and non-ESUS cases that did not meet strict ESUS criteria. ESUS was identified when the diagnostic evaluation showed a non-lacunar infarct without significant (≥50%) stenosis in arteries supplying the affected area, no major-risk cardioembolic sources, and no other determined causes [10].

Records were extracted for demographic data (sex, age, ethnicity [Europeans, Latin Americans, Asians, Black, Maghrebi]), clinical variables including vascular risk factors (arterial hypertension, obesity (defined as a body mass index [BMI] ≥30 kg/m^2^), diabetes, smoking), comorbidities (AF: atrial fibrillation prior or new onset, CKD: chronic kidney disease, VT: venous thrombosis, PFO: patent foramen oval), treatments (prior oral anticoagulants, antiplatelet therapy, statins, intravenous fibrinolysis, mechanical thrombectomy, subsequent antiplatelet or anticoagulant therapy), and laboratory findings (Factor VIII, protein C, protein S, antithrombin activity, anticardiolipin antibodies, anti-beta2-glycoprotein, lupus anticoagulant, D-dimer, total cholesterol, low-density lipoprotein cholesterol (LDL) cholesterol, C-reactive protein, homocysteine). Functional status was assessed using the modified Rankin Scale (mRS) at premorbid, discharge, and 3 months post-stroke, as evaluated by trained neurologists from the stroke unit. Neurological deficit was assessed on admission and at discharge using the National Institutes of Health Stroke Scale (NIHSS).

### 2.4 Statistical Analysis

Statistical analyses were conducted via SPSS version 27.0 (IBM Corp., Armonk, NY, USA). Continuous variables were tested for normality via the Shapiro‒Wilk and Kolmogorov‒Smirnov tests. Parametric variables are expressed as the mean and standard deviation (SD), nonparametric variables as the median and inter-quartile range (IQR), and categorical variables as frequencies.

Comparative analyses between groups were conducted using Fisher’s exact test or Pearson’s chi-square test for categorical variables. Independent *t*-tests, Mann-Whitney U test, Kruskal-Wallis test, and Spearman’s correlation were performed for continuous variables, as appropriate. A *p*-value of <0.05 was considered statistically significant. Boxplots were employed to visualize group differences and potential correlations. No corrections for multiple comparisons were made, since only one statistical test was performed for dichotomized Factor VIII and one for the continuous Factor VIII level. Consequently, adjustments for multiple comparisons were deemed unnecessary. Exploratory subgroup comparisons across TOAST etiological categories, as well as analyses treating Factor VIII as a continuous variable, were also conducted.

## 3. Results

**Fig. 1.**
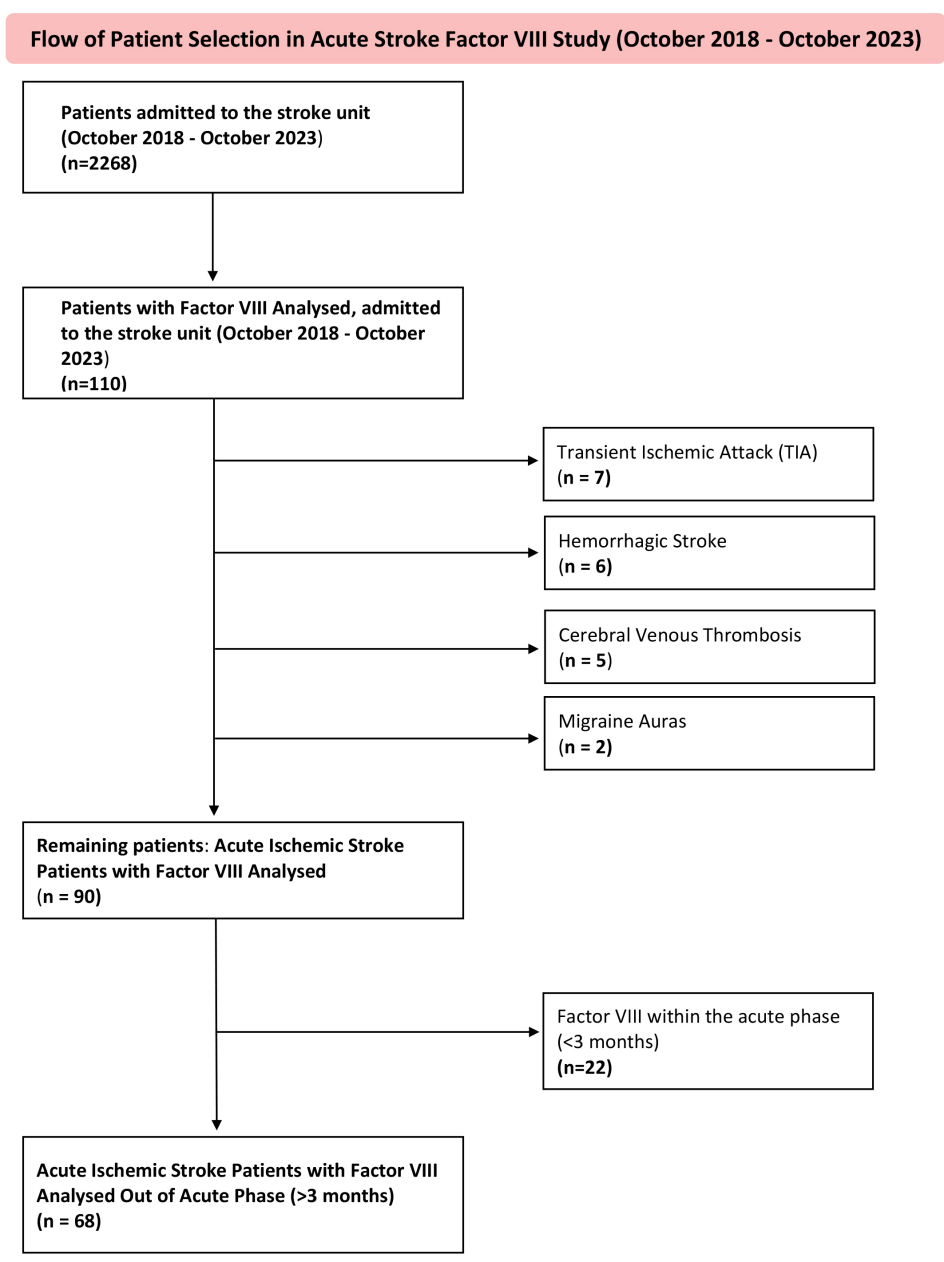
**Study flowchart**. The flowchart summarizes the patient selection process for the Acute Ischemic Stroke Factor VIII Study conducted between October 2018 and October 2023. During this period, the stroke unit registry recorded 2268 admissions (ischemic, hemorrhagic, and transient ischemic strokes, as well as stroke mimics). The study cohort was identified by cross-matching this registry with the Hematology Laboratory database of patients who had undergone thrombophilia screening, including Factor VIII testing. A total of 110 matched patients were identified. After excluding those with transient ischemic attack, hemorrhagic stroke, cerebral venous thrombosis, stroke mimics such as migraine aura (n = 20), and patients whose Factor VIII levels were measured during the acute phase (<3 months, n = 22), 68 patients with ischemic stroke evaluated outside the acute phase were included in the final study cohort.

During the study period, 2268 patients were admitted to the stroke unit. A total of 110 patients were identified by cross-matching the stroke unit registry with the Hematology Laboratory database of patients who had undergone thrombophilia screening, including Factor VIII testing. After applying the exclusion criteria (transient ischemic attack, hemorrhagic stroke, stroke mimics such as cerebral venous thrombosis or migraine aura, and Factor VIII measured during the acute phase), a total of 68 patients were included in the final analysis (Fig. 1).

The final cohort had a mean age of 50.28 years (SD 12.18), a male predominance (66.2%), and a majority of smokers (60.3%). The most common etiology was cryptogenic stroke (54.4%), followed by atherothrombotic (13.2%) and unusual (11.8%) causes. Among the unusual etiologies, four cases were related to coagulation disorders, one to a saccular aneurysm, one to a metabolic disorder, one to arterial dissection, and one to a carotid web.

The characteristics of patients in each group (Factor VIII ≤150% vs. >150%), as well as the bivariate analysis, are shown in Table 1. Demographic, clinical, laboratory, treatment, and stroke details were compared between patient groups with Factor VIII levels ≤150% (n = 40) and >150% (n = 28). Elevated Factor VIII (>150%) was not associated with the studied variables, including demographic characteristics, vascular risk factors, comorbidities, treatments, stroke details, or laboratory findings.

**Table 1.** **Baseline patient characteristics and results of bivariate analysis of normal versus elevated Factor VIII with remaining variables, and statistical analysis of factor continuous VIII levels**.

Variable	Total (n = 68)	F. VIII ≤150% (n = 40)	F. VIII >150% (n = 28)	*p*-value*	*p*-value**
Demographic characteristics					
Age, mean (SD)	50.28 (12.18)	48.59 (12.75)	50.48 (11.27)	0.68^¶^	0.18^¶⁢¶^
Male, n (%)	45 (66.20)	24 (60.00)	21.00 (75.00)	0.20^†^	0.45^§^
European, n (%)	63 (92.60)	38 (95.00)	25.00 (89.29)	0.63^†^	0.99^#^
Smoking (active/ex), n (%)	41 (60.20)	22 (55.00)	19.00 (67.86)	0.12^†^	0.31^#^
Comorbidities					
Obesity, n (%)	10 (14.70)	8 (20.00)	2 (7.14)	0.18^‡^	0.79^§^
Diabetes, n (%)	7 (10.29)	5 (12.50)	2 (7.14)	0.69^‡^	0.90^§^
HT, n (%)	26 (38.24)	16 (40.00)	10 (35.71)	0.72^†^	0.81^§^
Prior AF, n (%)	3 (4.41)	3 (7.50)	0 (0.00)	0.26^‡^	0.04^§^
New-onset AF, n (%)	1 (1.47)	0 (0.00)	1 (3.57)	0.41^‡^	0.11^§^
Prior VT, n (%)	6 (8.82)	2 (5.00)	4 (14.29)	0.22^‡^	0.19^§^
Laboratory					
Cholesterol, mean (SD)	159.47 (38.77)	159.38 (40.82)	162.64 (38.91)	0.35^¶^	0.67^¶⁢¶^
LDL, mean (SD)	97.16 (28.83)	95.82 (28.55)	99.36 (29.17)	0.35^¶^	0.75^¶⁢¶^
C-reactive protein, median (IQR)	0.20 (0.35)	0.22 (0.35)	0.14 (0.35)	0.42^§^	0.85^¶⁢¶^
Homocysteine, median (IQR)	12.80 (5.00)	12.90 (5.55)	12.50 (5.55)	0.71^§^	0.34^¶⁢¶^
Antithrombin, median (IQR)	106.60 (19.5)	106.40 (26.55)	108.00 (11.95)	0.13^§^	0.04^¶⁢¶^
Protein S, mean (SD)	96.76 (29.21)	95.62 (31.74)	96.61 (22.82)	0.95^¶^	0.17^¶⁢¶^
Protein C, median (SD)	107.92 (26.06)	106.85 (30.48)	109.44 (21.25)	0.86^¶^	0.61^¶⁢¶^
D-dimer, median (IQR)	327.00 (328.25)	282.50 (315.00)	350 (441.00)	0.98^§^	0.64^¶⁢¶^
Factor VIII, median (IQR)	142.35 (64.75)	116.55 (41.80)	187.85 (49.90)		
Altered Ab2, n (%)	2 (2.94)	2 (5.00)	0 (0.00)	0.50^‡^	0.09^§^
Altered cardiolipin Ac, n (%)	2 (2.94)	2 (5.00)	0 (0.00)	0.50^‡^	0.09^§^
Altered lupus ac, n (%)	7 (10.29)	5 (12.50)	2 (7.14)	0.69^‡^	0.40^§^
Treatments					
Fibrinolysis, n (%)	38 (55.88)	21 (52.50)	17 (60.71)	1.00^†^	0.79^§^
Thrombectomy, n (%)	15 (22.05)	9 (22.50)	6 (21.43)	0.92^†^	0.78^§^
Subsequent antiplatelet therapy, n (%)	60 (88.24)	36 (90.00)	24 (85.71)	0.71^‡^	0.30^§^
Subsequent anticoagulation, n (%)	10 (14.71)	6 (15.00)	4 (14.29)	1.00^‡^	0.80^§^
Stroke details					
Premorbid mRS, median (IQR)	0.00 (0.00)	0.00 (0.00)	0.00 (0.00)	0.37^§^	0.45^¶⁢¶^
mRS at discharge, median (IQR)	1.00 (2.00)	1.00 (2.00)	1.00 (2.00)	0.62^§^	0.62^¶⁢¶^
mRS at 3 months, median (IQR)	1.00 (2.00)	1.00 (2.00)	1.00 (2.00)	0.41^§^	0.85^¶⁢¶^
NIHSS on admission, median (IQR)	4.00 (7.50)	5.00 (7.00)	3.50 (6.75)	0.31^§^	0.30^¶⁢¶^
NIHSS at discharge, median (IQR)	1.00 (2.00)	1.50 (3.00)	0.00 (2.00)	0.37^§^	0.18^¶⁢¶^

SD, standard deviation; HT, hypertension; AF, atrial fibrillation; VT, venous thrombosis; IQR, inter-quartile range; mRS, modified Rankin scale; NIHSS, National Institutes of Health Stroke Scale. Percentages are calculated using the denominator of each subgroup (column percentage). Totals may not equal 100% due to rounding. Categorical variables were compared using the Pearson’s chi-square test (χ^2^ test)^†^, or Fisher’s exact test^‡^ when expected cell counts were <5. Statistical analysis for bivariate comparisons between dichotomized Factor VIII levels and categorical variables is shown in the first *p*-value column (*). Statistical analysis for continuous Factor VIII levels and other variables was performed using the Student’s *t*-test^¶^, Mann–Whitney U test^§^, Spearman’s correlation^¶⁢¶^, or Kruskal–Wallis test^#^, as appropriate (**). All *p*-values are uncorrected for multiple comparisons and are reported for exploratory purposes only. Factor VIII was the stratification variable; therefore, *p*-values are not applicable. LDL, low-density lipoprotein cholesterol.

Elevated Factor VIII levels >150% were not associated with the studied variables, including demographic characteristics, vascular risk factors, comorbidities, received treatments, stroke details, or laboratory findings.

**Fig. 2.**
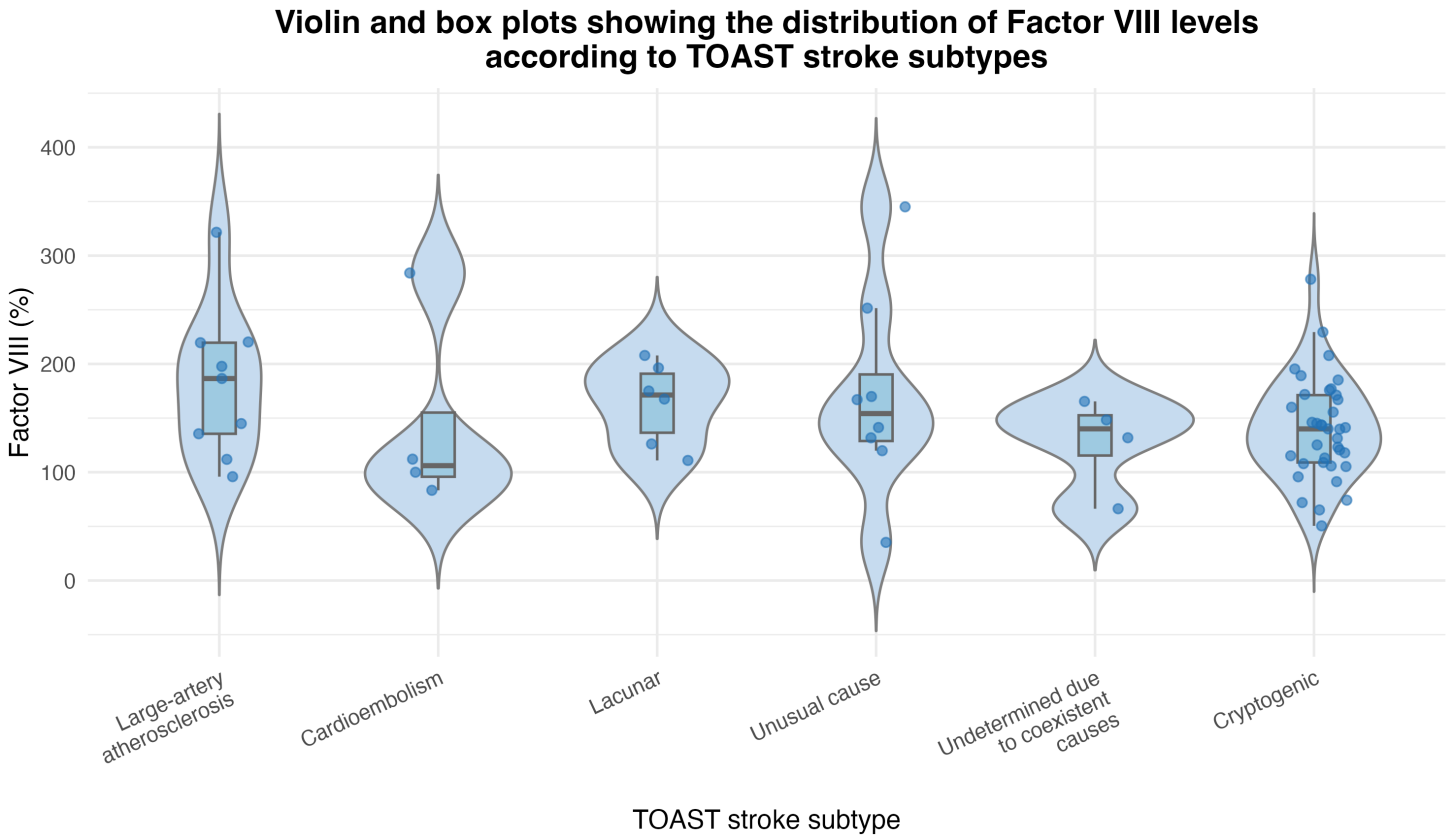
**Violin and box plots showing the distribution of Factor VIII levels according to TOAST stroke subtypes (large-artery atherosclerosis, cardioembolism, lacunar, unusual cause, undetermined due to coexistent causes, and cryptogenic)**. Each dot represents an individual observation (jittered for visibility). Although some variability was observed among subtypes, no statistically significant differences were detected (*p* = 0.38, Kruskal–Wallis test). The plots highlight the substantial overlap and the presence of mild outliers across etiological categories. TOAST, Trial of Org 10172 in Acute Stroke Treatment.

Elevated Factor VIII was observed in 41.2% of patients with ischemic stroke. No significant association was found between elevated Factor VIII and cryptogenic stroke (*p* = 0.27). Moreover, elevated Factor VIII did not differ significantly between the different stroke subtypes (*p* = 0.56) (Table 2). The distribution of Factor VIII across TOAST etiological categories is shown in Fig. 2. This illustrates a broad overlap and the presence of mild outliers.

**Table 2.** **Relationship between stroke subtypes and Factor VIII levels as a continuous and dichotomized variable (≤150% and >150%). Stroke subtypes were dichotomized to analyse the association of F. VIII levels**.

Stroke subtype, n (%)	F. VIII % value, median (IQR)	Statistical significance (*p*)	F. VIII ≤150%, n (%)	F. VIII >150%, n (%)	Statistical significance (*p*)
Large-artery atherosclerosis, 9 (13.2)	186.50 (96.25)	0.12	4 (44.40)	5 (55.60)	0.47
Cardioembolism, 4 (5.9)	106.05 (153.55)	0.35	3 (75.00)	1 (25.00)	0.45
Lacunar, 6 (8.8)	171.30 (76.88)	0.30	2 (33.30)	4 (66.70)	0.19
Unusual cause, 8 (11.8)	154.10 (108.23)	0.53	4 (50.00)	4 (50.00)	0.43
Coexistent causes, 4 (5.9)	140.05 (78.45)	0.56	3 (75.00)	1 (25.00)	0.45
Cryptogenic, 37 (54.4)	140.00 (63.05)	0.19	24 (64.90)	13 (35.10)	0.27

For the overall cohort, the median Factor VIII level (expressed as a percentage) was 142.35 (IQR: 64.75). The median (IQR) value for each subtype was as follows: large-artery atherosclerosis 186.5% (96.25), cardioembolism 106.05% (153.55), lacunar 171.3% (76.88), unusual causes 154.1% (108.23), coexistent causes 140.05% (78.45), and cryptogenic stroke 140% (63.05). No statistically significant differences were observed between the subtypes (*p* = 0.38) (Table 2, Fig. 2).

**Fig. 3.**
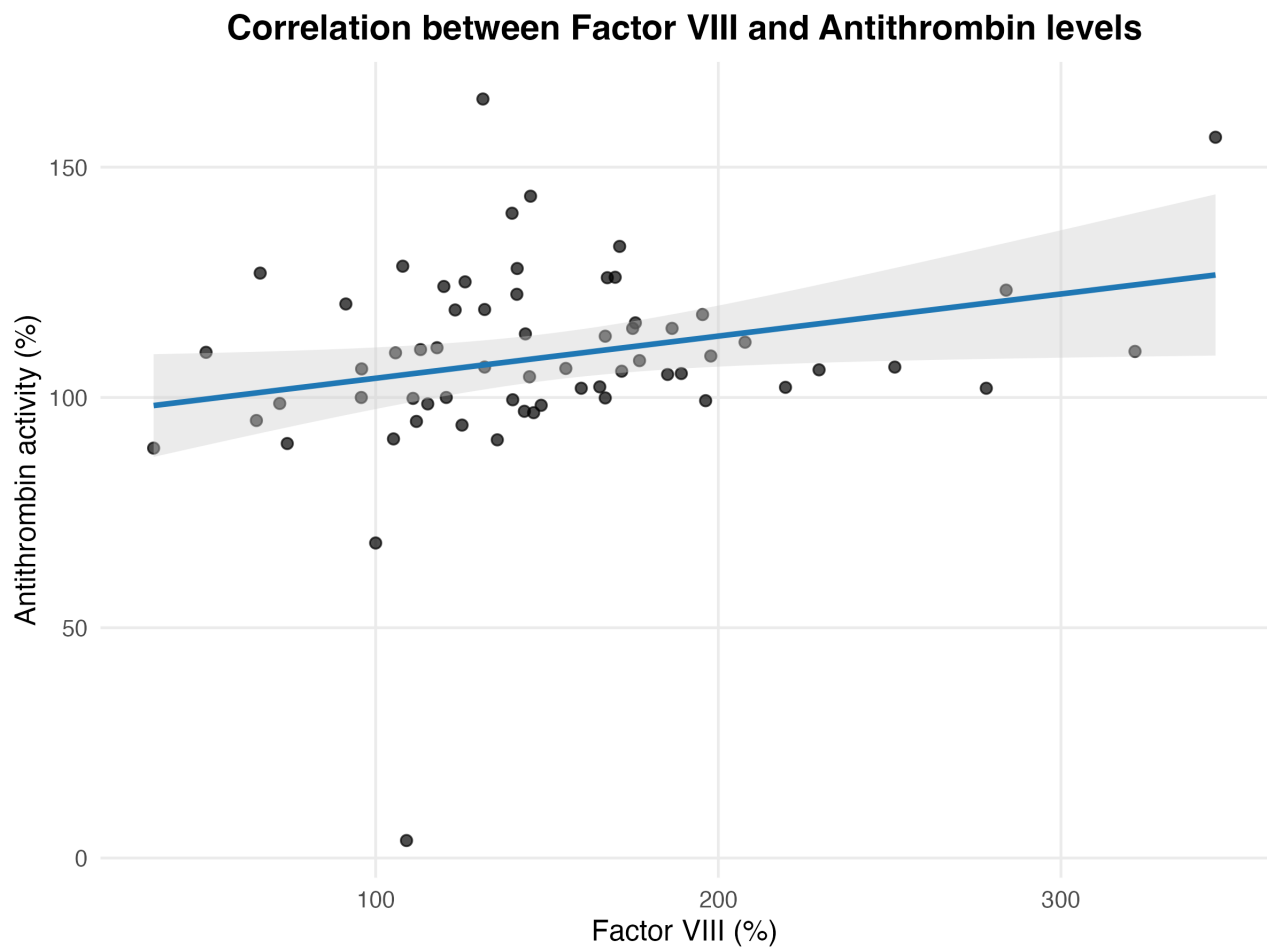
**Scatter plot illustrating the relationship between Factor VIII levels and antithrombin activity in patients with ischemic stroke**. Each point represents an individual patient. The blue line shows the fitted linear regression with its 95% confidence band (shaded area). A weak but statistically significant positive correlation was observed (Spearman’s ρ = 0.26, *p* = 0.039), indicating that higher Factor VIII levels were associated with slightly increased antithrombin activity.

A significant correlation was observed between Factor VIII level and antithrombin activity (Spearman’s ρ = 0.26, *p* = 0.039). This remained consistent using Kendall’s τ (τ = 0.18, *p* = 0.034), supporting the robustness of this finding. The relationship between these variables is illustrated in Fig. 3, showing the fitted regression line with the 95% confidence band.

The various demographic factors, vascular risk factors, comorbidities, treatments, and laboratory findings were also evaluated in relation to Factor VIII levels (Table 1). A significant association was observed between the Factor VIII level and prior AF (*p* = 0.04). The boxplot for this comparison is shown in **Supplementary Fig. 1**. Visual inspection confirmed that neither association was driven by outliers, although the small number of patients with prior AF (n = 3) warrants cautious interpretation. No other statistically significant associations or correlations with Factor VIII were observed with the remaining variables.

## 4. Discussion

The current study was conducted on patients admitted to a stroke unit, in whom the Factor VIII level was determined as part of the routine etiological assessment. To avoid increases due to the acute phase, Factor VIII evaluation was performed at least 3 months after the stroke, given that it acts as an acute-phase reactant. Elevated Factor VIII levels were found in 41.2% of patients. No significant association was found between elevated Factor VIII and cryptogenic stroke, which was the most common subtype in this cohort. Additionally, Factor VIII levels showed a weak correlation with antithrombin levels and a significant association with prior atrial fibrillation.

Chang *et al*. [5] reported elevated Factor VIII in 72.4% of patients with ischemic stroke, finding an association between higher levels and greater stroke severity, but not with stroke etiology. In their study, Factor VIII was measured during the acute phase, and no subsequent follow-up was performed. Karttunen *et al*. [9] conducted a case-control study to identify risk factors for cryptogenic ischemic stroke. They found that increased Factor VIII levels were associated with this type of stroke, and emphasized the need for further research to confirm their findings. The discrepancy between our findings and those of Karttunen *et al*. [9] may be due to several methodological and population differences. The study by Karttunen *et al*. [9] focused on young adults (15–60 years) with cryptogenic stroke and excluded cardioembolic sources and patent foramen ovale. In contrast, our cohort included an unselected real-world cohort of ischemic stroke patients with a broader age range, various etiologies, and more vascular comorbidities. The nature of their sample may have increased the ability to detect subtle associations, whereas our retrospective approach and smaller sample size may have reduced statistical power. Differences in ethnicity, genetic background, sex distribution, and laboratory assay methods may also contribute to the differing results.

The MEGASTROKE Consortium [7] employed Mendelian randomization to investigate genetic associations between 670,000 stroke cases and various factors (traits) involved in hemostasis. An association was found between elevated antigenic Factor VIII and stroke risk in subjects with atrial fibrillation. The present study also observed a significant association between Factor VIII levels and prior AF, although the small number of AF patients limits the significance of this result. Finally, Siegler *et al*. [8] reviewed the association between stroke etiology and Factor VIII, finding a significant association with large-vessel atherothrombotic strokes in measurements performed within the first 3 months post-stroke [11].

Several reasons may explain the contradictory results between these studies. Elevated levels of Factor VIII have been associated with chronic inflammation, liver disease, malignancy, renal disease, hyperthyroidism, intravascular hemolysis, obesity, diabetes, hypertriglyceridemia, age, pregnancy, and postsurgical state [4, 12]. Elevated Factor VIII levels have also been detected in 11% of the general population [13]. Many of these variables persist 3 months after the stroke event and were not accounted for in earlier studies, which could explain the inconsistent results and lack of reliable associations with a specific stroke subtype. Nonetheless, it is important to understand the pathophysiological role of Factor VIII in stroke patients, considering previous reports of persistently elevated Factor VIII in subjects with ischemic stroke [6, 14, 15].

Although elevated Factor VIII has been hypothesized as a common thrombogenic factor in several subtypes of stroke, the mechanism by which it increases thrombogenicity and the risk of thrombotic events remains unclear. This nonspecific mechanism may be related to the well-known relationship between inflammation, chronic or acute events, and thrombotic events, such as those occurring with the Severe Acute Respiratory Syndrome Coronavirus 2 (SARS-CoV-2) virus, which has also been linked to Factor VIII [16]. Elevated Factor VIII levels may act as a marker of inflammation or ongoing coagulation rather than constituting a direct causal factor, reflecting chronic endothelial activation or systemic inflammatory states that accompany vascular disease.

Rohmann *et al*. [6] studied Factor VIII and other coagulation factors in a cohort of 576 patients with ischemic stroke (31% of undetermined origin). These workers reported that increased levels of Factors VIII and XI after 3 years were related to a new ischemic vascular event. Gouse *et al*. [17] also reported an increased risk of recurrence in patients with elevated Factor VIII.

Differing results between studies are likely to reflect methodological heterogeneity. As summarized in **Supplementary Table 1**, prior investigations vary in design, sample size, timing of Factor VIII measurement, and adjustment for confounders. Acute-phase studies may overestimate Factor VIII due to its reactive behavior, whereas post-acute assessments, as in the present study, reflect more stable levels. These methodological variations may explain the inconsistent associations reported in the literature.

Other serum biomarkers have been extensively studied in ischemic stroke. Among them are cardiac biomarkers in peripheral blood, such as B-type natriuretic peptide and N-terminal pro-brain natriuretic peptide (NT-proBNP), which have been proposed as indicators of cardioembolic ischemic stroke [18]. Coagulation biomarkers can also help to identify certain causes of ischemic stroke, of which D-dimer is the most extensively studied. D-dimer is an inflammatory marker and a product of fibrin degradation. It is sometimes combined with other markers of hemostatic activation in patients with occult neoplasia or other hypercoagulable states [19]. Factor VIII was shown to be correlated with D-dimer, mainly in black individuals, and this correlation could not be explained by other clinical or socioeconomic factors [20]. However, D-dimer did not correlate with Factor VIII in the present study, and only antithrombin levels outside the normal range were correlated with elevated Factor VIII levels.

Recently, there has been growing interest in biomarkers based on RNA expression in peripheral red blood cells as an aid in the diagnosis of stroke, its causal subtype [21], and its prognosis [22, 23]. This technique is based on the differential gene expression of RNA according to the type of stroke and its etiology, providing insights into the immune response, inflammatory response, and post-stroke angiogenesis [21, 23].

Our study has several limitations. First, the relatively small sample size reduced our ability to detect differences between groups and limited the statistical power of the analysis. Although the sample size limited the feasibility of formal sensitivity analyses, several exploratory approaches were conducted to assess the robustness of the findings. Comparisons of Factor VIII across TOAST etiological subgroups, as well as analyses treating Factor VIII as a continuous variable, did not reveal trends suggesting a specific association with cryptogenic stroke. Moreover, the significant association observed with prior atrial fibrillation—despite the small size of this subgroup—indicates that the study was able to detect clinically relevant associations when present. While exploratory in nature, these analyses provide complementary support that the lack of association between elevated Factor VIII and cryptogenic stroke is unlikely to be attributable to an unstable or atypical distribution within the cohort. This limitation stems from the inclusion criteria, which reflect real-world clinical practice for patients admitted to the stroke unit and who undergo thrombophilia testing with Factor VIII measurement. In particular, there was an increased risk of type II error (false negatives) due to the small number of patients with elevated Factor VIII (n = 28), meaning that modest or clinically relevant associations may not have been detected. Future studies with larger, prospectively recruited cohorts and more balanced group sizes are warranted to confirm our findings and improve the precision of estimates regarding the association between Factor VIII and stroke subtypes.

Second, the retrospective cross-sectional study design and inclusion bias with a high percentage of cryptogenic strokes may limit the generalizability of our results. Factor VIII testing was performed at the discretion of the treating physician rather than applied systematically. Consequently, the study sample may over-represent patients who were younger, exhibited atypical stroke presentations, lacked conventional vascular risk factors, or were suspected of having an underlying hypercoagulable state. This selective inclusion could increase the proportion of cryptogenic strokes within the cohort and may either attenuate or exaggerate the apparent association between elevated Factor VIII levels and this subtype. Although the direction and magnitude of this potential bias cannot be quantified, acknowledging its possible influence is essential for interpreting the observed lack of association. Furthermore, although some thrombophilia markers (e.g., fibrinogen, Factor VII, factor XI, Factor V Leiden, lipoprotein (a), prothrombin G20210A, methylenetetrahydrofolate reductase [MTHFR], etc.) were tested in part of the cohort, these variables were not systematically available for all patients and hence were not included in the statistical analysis. This may have limited the completeness of the thrombophilia evaluation.

Third, given the limited sample size, multivariable adjustment was not performed for potential confounders known to influence Factor VIII levels, such as age, inflammation, obesity, smoking, liver, and thyroid disease. Future studies should incorporate adjusted models to clarify the independent contribution of Factor VIII to ischemic stroke risk and subtypes.

Finally, as multiple comparisons were performed, the possibility of type I error inflation cannot be ruled out, and *p*-values should be interpreted as exploratory.

Despite these limitations, the trend observed for elevated Factor VIII in a high percentage of non-cryptogenic strokes suggests this may not be exclusive to a single type of cryptogenic stroke. We also highlight the innovative approach of this study in addressing ischemic stroke from a thrombophilia perspective. Larger studies may be needed to better understand the complex relationship between Factor VIII and ischemic stroke, as well as genetic and racial factors that may interact with Factor VIII levels to affect outcomes [23, 24]. It has been reported that 57% of the total variation in Factor VIII levels is genetically determined [25].

## 5. Conclusions

The possible prothrombotic role of Factor VIII in ischemic stroke has been suggested in previous studies. Factor VIII levels were elevated in 41.2% of patients in our sample, but showed no significant association with this subtype of stroke. Further research is warranted to clarify whether elevated Factor VIII is an independent factor associated with specific etiological types of stroke, or is simply a consequence of inflammatory events, their systemic repercussions, or other unknown factors.

## Availability of Data and Materials

The datasets generated and analyzed during the current study are available from the corresponding author on reasonable request.

## Supplementary Material

Supplementary material associated with this article can be found, in the online version, at https://doi.org/10.31083/RN44168.
