# Supplementary material for: Influence of Coagulation Factor VIII on Ischemic Stroke
Source: Rev Neurol. 2026 Jan 26;81(1):44168. doi: 10.31083/RN44168 (PMC12873674; doi:10.31083/RN44168)

**SUPPLEMENTARY MATERIAL**

**Supplementary Table 1. Summary of previous studies evaluating Factor VIII levels and ischemic stroke.**

| **Study (Year)** | **Design** | **Sample size** | **Timing of Factor VIII measurement** | **Main finding(s)** | **Adjustment for confounders** |
| --- | --- | --- | --- | --- | --- |
| **Chang et al. (2014)** [5] | Observational, acute-phase study | 232 | Within 72 h after stroke onset | Elevated FVIII associated with greater stroke severity, but not with etiology | No adjustment |
| **Karttunen et al. (2002)** [9] | Case-control study (cryptogenic stroke vs. controls) | 125 | >3 months after event | Elevated FVIII associated with cryptogenic stroke | Adjusted for age, sex, and vascular risk factors |
| **Siegler et al. (2015)** [11] | Cross-sectional study | 138 | ≤3 months post-stroke | Higher FVIII in large-artery atherosclerosis vs. small-vessel stroke | Partially adjusted |
| **Rohman et al. (2019)** [6] | Prospective cohort study | 576 | 3 years post-stroke | Elevated FVIII and XI associated with recurrent ischemic events | Adjusted |
| **Gouse et al. (2020)** [17] | Prospective cohort study | 218 | Mean follow-up 2 years | Elevated FVIII associated with higher recurrence risk | Adjusted |
| **Present study (2023)** | Retrospective observational study | 68 | ≥3 months post-stroke | Elevated FVIII in 41.2% of patients; no association with cryptogenic stroke | Non adjustement (limited by sample size) |

**Supplementary Fig. 1.** Boxplot showing Factor VIII levels according to prior atrial fibrillation status. The “Prior AF” group included three patients, explaining the narrow range of values.


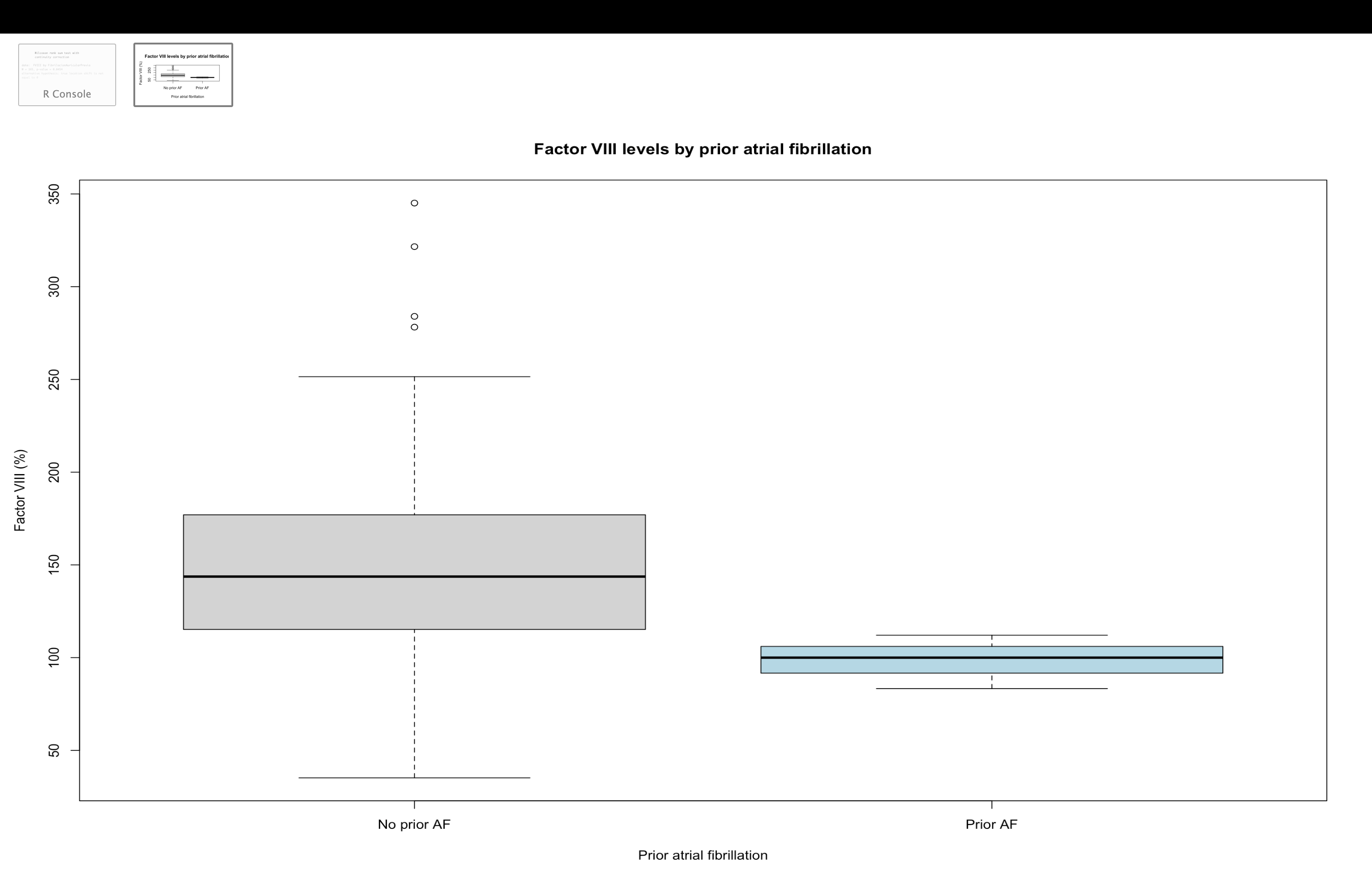

Supplement: Supplementary file 1 [file 1576-6578-81-1-44168-s1.docx]
